# The impact of erector spinae plane block on neutrophil-to-lymphocyte ratio and postoperative nausea and vomiting in lumbar spine surgery patients: a protocol for a randomized controlled trial

**DOI:** 10.3389/fmed.2025.1630821

**Published:** 2025-08-28

**Authors:** Jia-jun Wang, Yan Wang, Xue-Fei Li, Song-Song Chen, Yujie Wang, Peng-yu Sun, Hai-Kun Yang, Pengcai Shi, Guanghan Wu

**Affiliations:** ^1^Department of Anesthesiology, The First Affiliated Hospital of Shandong First Medical University (Shandong Provincial Qianfoshan Hospital), Shandong Institute of Anesthesia and Respiratory Critical Care, Jinan, Shandong, China; ^2^School of Anesthesiology, Shandong Second Medical University, Weifang, Shandong, China; ^3^Department of Nursing, The First Affiliated Hospital of Shandong First Medical University and Shandong Provincial Qianfoshan Hospital, Jinan, Shandong, China; ^4^Department of Special Examination, The Second Affiliated Hospital of Shandong University of Traditional Chinese Medicine, Jinan, Shandong, China; ^5^Shandong First Medical University (Shandong Academy of Medical Sciences), Jinan, Shandong, China; ^6^Department of Anesthesiology, Qingzhou People's Hospital, Weifang, Shandong, China

**Keywords:** neutrophil to lymphocyte ratio (NLR), postoperative nausea and vomiting (PONV), erector spinae plane block (ESPB), postoperative analgesia, lumbosacral spine surgery

## Abstract

**Background:**

Lumbar spine surgery is associated with significant postoperative pain and a high incidence of postoperative nausea and vomiting (PONV). Inflammation is a known contributor to PONV risk, and the neutrophil-to-lymphocyte ratio (NLR) is a cost-effective parameter for evaluating systemic inflammation. Erector spinae plane block (ESPB) under ultrasound guidance is a regional anesthesia technique that may reduce postoperative pain, inflammatory responses, and opioid consumption. However, evidence on the relationship between preoperative NLR, PONV, and the effects of ESPB is limited.

**Overview:**

This prospective, double-blind, single-center, parallel-group study will enroll 220 patients undergoing elective lumbar spine surgery under general anesthesia. Patients will be stratified by a preoperative NLR threshold of 2 into two equal groups and further randomized to receive either ultrasound-guided ESPB with ropivacaine or a saline control after anesthesia induction. All participants will receive standard PONV prophylaxis with intravenous ondansetron. Primary endpoints include the incidence of nausea, vomiting, and antiemetic requirements in the first and second 24-hour postoperative periods, as well as postoperative NLR. Secondary endpoints include pain scores, intraoperative anesthetic consumption, total postoperative analgesic use, time to first analgesic pump activation, patient satisfaction, recovery times, length of stay, opioid-related side effects, and serum neutrophil extracellular traps.

**Results:**

At the time of submission, the trial is ongoing and in the patient recruitment phase. No results are yet available.

**Discussion:**

The study is designed to evaluate whether preoperative NLR can serve as a biomarker for PONV and to determine the effect of ESPB on NLR, PONV, and postoperative recovery parameters in lumbar spine surgery patients. The findings may provide evidence for individualized PONV prevention strategies and the perioperative application of ESPB.

**Conclusion:**

This trial will clarify the predictive value of NLR for PONV and assess the efficacy of ESPB in modulating postoperative inflammation and improving recovery in lumbar spine surgery.

**Clinical trial registration:**

ClinicalTrials.gov identifier, NCT06127966.

## Highlights

- This study employs rigorous screening criteria, a random allocation scheme, and a two-arm randomized controlled trial for four group.
- This study presents a rigorous method for comparing the preoperative neutrophil-to-lymphocyte ratio to PONV and the effect of erector spinae plane block on NLR and PONV in patients undergoing lumbar spine surgery.
- This study is conducted at a single center, and the findings may not apply to a broader population. A single-center design may limit the external generalizability of the findings. We plan to conduct future multicenter studies to validate the applicability and robustness of our results.

## 1 Introduction

The incidence of lumbar spine disease significantly increases as the population ages and lifestyles change. The number of patients with lumbar spine disease in China exceeds 200 million, with lumbar disk herniation accounting for 15.2% of the total and showing a gradual upward trend over the years. Posterior lumbar fixation procedures are mainly indicated in patients with spinal instability or degenerative lumbar stenosis, rather than in those with simple lumbar disk herniation; however, spinal surgery often involves significant trauma and the use of high doses of opioids and anesthetics, resulting in a high incidence of complications such as severe postoperative pain and nausea and vomiting. If these complications are not effectively controlled, they can affect postoperative recovery and extend the length of hospital stay.

Postoperative nausea and vomiting (PONV) are common complications (17–20). In patients undergoing lumbar surgery, PONV can lead to further complications. The most common consequence is an extended hospital stay. More severe complications can include implant displacement, aspiration, pulmonary infection, and spinal cord injury (1–3). Although PONV is generally self-limiting and nonfatal, it significantly increases the risk of dehydration, electrolyte imbalance, suture line rupture, hypertension, bleeding, esophageal rupture, and life-threatening airway obstruction. PONV can prolong post-anesthesia care unit (PACU) stay and overall hospitalization time, decrease patient satisfaction, lead to unexpected readmissions, and increase hospital costs. Therefore, preventing and managing PONV are crucial for improving patient outcomes and reducing healthcare costs (4).

The first step in PONV prevention is identifying risk factors and high-risk patient groups. Many anesthesia-related risk factors contribute to PONV, including anesthesia techniques, volatile anesthetics use, nitrous oxide use, anesthesia duration, opioid use, and surgery type (5). Inflammation increases the risk of PONV; the neutrophil-to-lymphocyte ratio (NLR) (21, 22) is a cost-effective parameter for diagnosing and monitoring systemic inflammatory diseases (6, 7). Severe vomiting during pregnancy is related to NLR, but research exploring the potential relationship between PONV and NLR is limited. Although PONV prevention increases healthcare costs, it costs less than treating PONV and the associated complications. Therefore, establishing evidence-based parameters for identifying patients who require preoperative PONV prevention is important for reducing hospital costs.

Lumbar spine surgery is a painful surgical procedure. Postoperative pain is classified as acute primarily because of the physiological stress response triggered by surgical trauma and inflammation. Ultrasound-guided erector spinae plane block (ESPB) is a new local anesthetic technique that blocks the dorsal and ventral branches of the thoracolumbar spinal nerves. It is used in thoracic, abdominal, breast, urological, weight loss, and hip joint surgeries for combined anesthesia and postoperative pain control. Under ultrasound guidance, ESPB provides precise localization, accurate drug administration, effective pain relief, and a minimal effect on the patient’s overall condition, with no apparent adverse reactions (8–13). However, its application in lumbar spine surgery is limited. Reports suggest this technique can also be used in lumbar spine surgery.

Performing ESPB during lumbar–sacral surgery significantly reduces postoperative pain scores, decreases patient stress responses, lowers inflammatory reactions, and reduces the dosage of opioid medications and their immunosuppressive and other side effects (8–13). Inflammatory mediators may influence the nausea and vomiting center by entering the circulation. Neutrophils are key effectors of sterile inflammation; increased and activated neutrophils form neutrophil extracellular traps (NETs) after acute trauma. NETs are involved in the response to various infectious and non-infectious stimuli. Following surgical trauma, increased NET formation may amplify systemic inflammatory responses and promote the release of proinflammatory cytokines. These mediators can sensitize the chemoreceptor trigger zone (CTZ) and the vomiting center in the brainstem, thereby increasing the risk of PONV. Ultrasound-guided erector spinae plane block (ESPB) may attenuate this inflammatory cascade through sympathetic blockade and reduction of nociceptive inputs. By decreasing the surgical stress response, ESPB could lower neutrophil activation and consequent NET formation, thereby modulating postoperative inflammatory burden. However, reports on whether ESPB affects NLR, NETs, and PONV are limited.

Therefore, this study aims to investigate whether preoperative NLR is a biomarker for PONV and explore the effect of ESPB on NLR and PONV.

### 1.1 Objective

The primary objective of this study is to investigate the effects of ESPB on NLR and PONV. The secondary objectives are to explore whether preoperative NLR can serve as a biomarker for PONV and to examine the analgesic effects of ESPB in spinal surgery and its influence on opioid medication dosage.

## 2 Methods and analysis

### 2.1 Study design

This prospective, single-center, double-blind, randomized controlled study was conducted at the First Affiliated Hospital of Shandong First Medical University, a tertiary university hospital in China. The study is registered with ClinicalTrials.gov (NCT06127966) and is scheduled to commence on November 1, 2023, with an expected completion time of 2 years. The study protocol adhered to the Intervention Trial Recommendation Statement and Uniform Standard Statement for Reporting Trials to ensure accurate reporting of trial results. We used the SPIRIT reporting guidelines (14).

### 2.2 Study setting

This study was performed at The First Affiliated Hospital of Shandong First Medical University in Jinan City, Shandong Province.

### 2.3 Patients and recruitment

**Figure 1:**
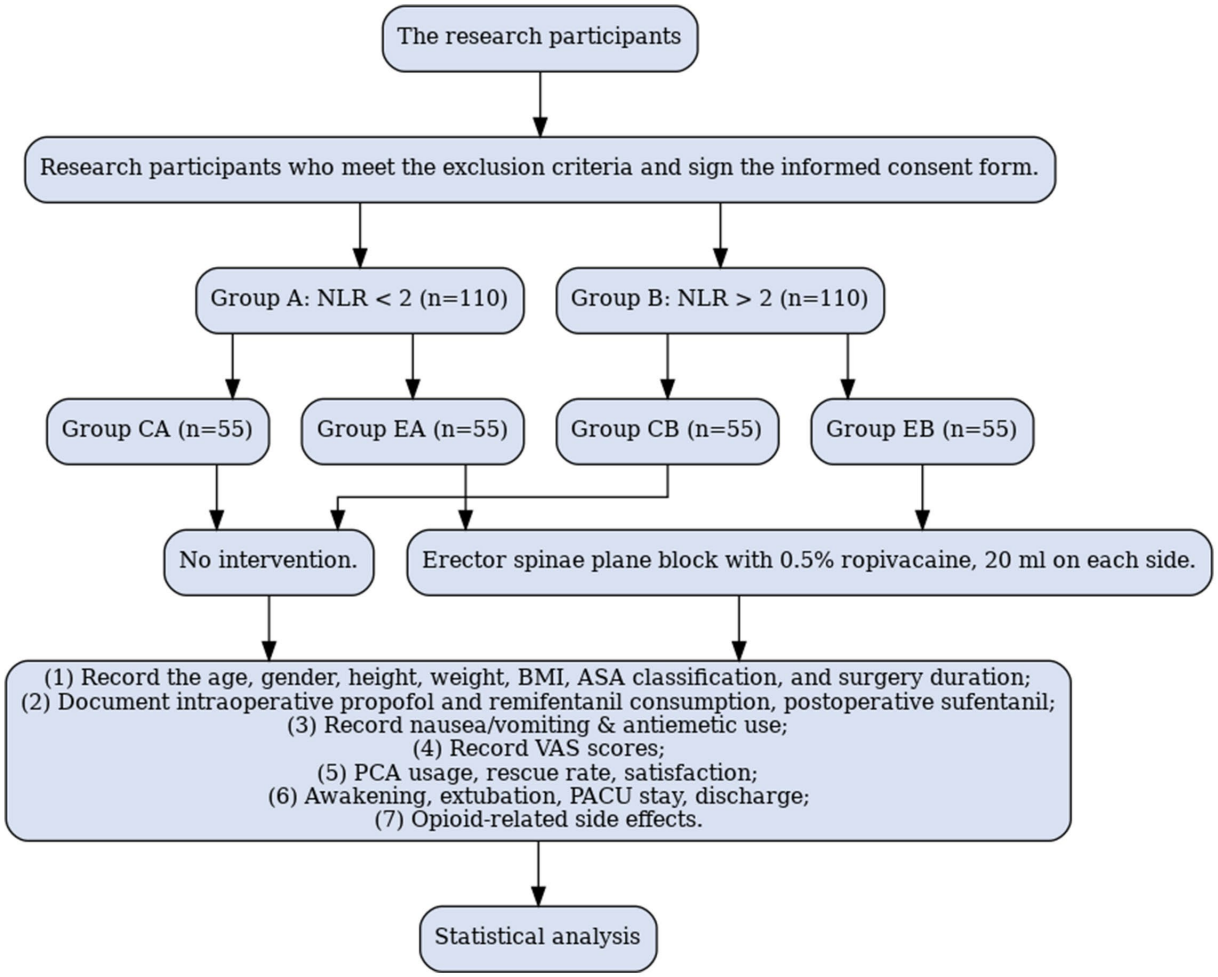
Study flow chart.

The study will include 220 patients aged 18–80 years, classified as American Society of Anesthesiologists (ASA) grade I to III planning to undergo elective prone lumbar spine surgery under general anesthesia from August 1, 2025, to September 1, 2026. Each patient will provide written informed consent and be assigned randomly to a different group based on the inclusion and exclusion criteria. All patients participating in the trial were given 3 to 5 days to learn about the study before signing the informed consent form. Preoperatively, the NLR was calculated by dividing the neutrophil count by the lymphocyte count in the complete blood count. A preoperative NLR threshold value of 2 (23–29) was accepted; the patients were divided into two groups based on values above and below 2; each group comprised 110 patients. Patients with a preoperative NLR < 2 are included in Group A (*n* = 110); those with a preoperative NLR > 2 are included in Group B (*n* = 110). Using a random number table, Group A is divided into an ESPB (EA group, *n* = 55) and a control group (CA group, *n* = 55); Group B is divided into an ESPB (EB group, *n* = 55) and a control group (CB group, *n* = 55). Randomization is achieved using a computer-generated table of random numbers (Figure 1).

### 2.4 Inclusion criteria

The inclusion criteria are patients aged 18–80 years scheduled for elective prone lumbar spine surgery under general anesthesia, specifically including one- or two-level posterior lumbar decompression, discectomy, or posterior lumbar interbody fusion (PLIF) for degenerative conditions. These patients must have an ASA classification of grade I to III and sign informed consent forms to participate in this clinical study.

### 2.5 Exclusion criteria

The exclusion criteria include preoperative blood transfusion, uncontrolled systemic diseases, patients with known uncontrolled systemic inflammatory diseases (e.g., rheumatoid arthritis, lupus, or active infections), gastrointestinal system disorders, history of antiemetic (15) and anticholinergic drug use, history of adverse reactions related to surgery, deformity correction surgeries, defined as procedures involving instrumentation across three or more levels or aimed at correcting scoliosis or kyphosis, infection at the puncture site, coagulation disorders, long-term use of sedatives and analgesics before surgery, psychiatric disorders, language communication barriers, allergy to ropivacaine, involvement in other clinical studies in the last 3 months, history of previous lumbar surgery, and unwillingness to participate in this study.

### 2.6 Intervention

For patients undergoing posterior lumbar spine surgery under general anesthesia, a researcher will assess whether the patient meets this study’s inclusion and exclusion criteria 1 day before the surgery. The participants who meet the inclusion criteria must sign an informed consent form after being fully informed of the study procedures, potential benefits, and risks. Preoperatively, the NLR will be calculated by dividing the neutrophil count by the lymphocyte count. The pre-calculated NLR threshold of 2 will be accepted. Patients with a preoperative NLR < 2 will be included in Group A (*n* = 110); those with a preoperative NLR > 2 will be included in Group B (*n* = 110). Using a random number table, Group A will be divided into ESPB (EA group, *n* = 55) and control groups (CA group, *n* = 55); Group B will be divided into ESPB (EB group, *n* = 55) and control groups (CB group, *n* = 55). The researcher will allocate the drugs using an envelope method to conceal the randomization sequence; a specially trained anesthesia nurse will deliver the implementation plan to the corresponding operating room before surgery.

1. ESPB Group (EA group + EB group): Routine anesthesia induction, followed by ultrasound-guided bilateral ESPB with 0.5% ropivacaine (20 mL) after anesthesia induction.
2. Control Group (CA group + CB group): Routine anesthesia induction, followed by ultrasound-guided bilateral ESPB injection with 0.9% saline (20 mL) after anesthesia induction.

Upon entering the operating room, standard intravenous access will be established; noninvasive blood pressure, electrocardiogram, and oxygen saturation (SpO_2_) will be monitored. Considering that most patients undergoing this surgery are older and positioned prone, radial artery catheterization will be performed using the FloTrac sensor to enable invasive blood pressure monitoring and ensure patient safety. All patients will receive total intravenous anesthesia.

With the patient in the supine position, a face mask will be used for oxygenation and denitrogenation for 3 min before anesthesia induction. Anesthesia induction will include midazolam (0.02 mg/kg), sufentanil (0.2–0.3 μg/kg), propofol (1.5–2 mg/kg), and atracurium (0.6 mg/kg). After the disappearance of the eyelash reflex and jaw relaxation, tracheal intubation will be performed using a visual laryngoscope, and the endotracheal tube will be secured. Mechanical ventilation will be initiated by adjusting respiratory parameters.

After positioning the patient in the prone position, the appropriate lumbar vertebral level will be identified based on preoperative surgical markings using either surface landmarks or ultrasound. The ESPB will be performed bilaterally under ultrasound guidance based on the planned surgical levels. For single-level lumbar surgery (e.g., L3–L4), the block is administered at the transverse process of the upper vertebra (e.g., L3). For two-level surgery (e.g., L3–L5), the block is performed at the middle level (e.g., L4) to ensure optimal spread of local anesthetic. The skin at the puncture site will be disinfected with iodine. An ultrasound low-frequency convex array probe with a sterile sheath will be placed in the parasagittal direction, 3 cm lateral to the midline. The lumbar transverse process, overlying erector spinae, and latissimus dorsi muscles will also be identified. The needle will be advanced in-plane; upon contact with the bony transverse process, confirmation of the correct needle position will be obtained by aspirating 2–3 mL of isotonic saline for hydrodissection. In both EA and EB groups, 20 mL of 0.5% ropivacaine will be administered on each side for the lumbar transverse process surface, providing bilateral ESPB. In the CA and CB groups, 20 mL of 0.9% saline will be administered on each side under ultrasound guidance to the lumbar transverse process surface, serving as a control.

Anesthesia maintenance will utilize propofol (4–6 mg/kg/h) and remifentanil (12–20 μg/kg/h), targeting a BIS range of 40–60. Blood pressure and heart rate will be maintained within 20% of the preoperative baseline values, with adjustments made according to surgical needs. Atracurium will be added intermittently to maintain muscle relaxation. Ten minutes before the end of the surgery, the infusion of anesthetic drugs will be stopped; nonsteroidal anti-inflammatory drugs will not be administered to any patient before the incision or at the end of the surgery. If blood pressure and heart rate variations exceed 20% of the preoperative values, the corresponding medications (5 mg ephedrine, 0.2 mg atropine, 20 mg esmolol, or 0.5 mg nitroglycerin) will be administered promptly. Considering that glucocorticosteroids, such as dexamethasone, may affect the NLR values, they were not considered preoperatively or postoperatively (16).

All patients will receive a standardized 48-h postoperative patient-controlled intravenous analgesia (PCIA) regimen to ensure consistency in analgesic management and allow for objective assessment of analgesic efficacy and opioid consumption. The PCIA formulation will consist of 2 μg/kg sufentanil, 16 mg ondansetron, and 0.9% saline to a total volume of 100 mL. The PCIA device will have a background infusion rate of 2 mL/h, a bolus dose of 2 mL, and a lockout interval of 15 min with no background dose. The patients will be transferred to the PACU for postoperative recovery. If the Visual Analogue Scale (VAS) score at rest is ≥4, the patient or nurse anesthetist will press the PCIA button to administer the drug until the VAS score is <4. If the VAS score remains >4 after three consecutive presses (15 min between presses), the PCIA pump may be gradually adjusted upward in a single dose on demand until the patient’s VAS score is <4. After the pain episodes finish, the PCIA settings will be readjusted to the original parameters. Pain assessments will be conducted every 6 h. Postoperative follow-up data will be collected by designated personnel.

### 2.7 Data collection and management

Demographic and clinical data will be collected from all patients, including age, sex, height, weight, body mass index, ASA classification, surgery duration, the intraoperative consumption of drugs, such as propofol and remifentanil, and total analgesic consumption in the first and second 24-h periods postoperatively. Nausea, vomiting, and antiemetic requirements will be recorded in the PACU for the first and second 24-h periods postoperatively. The VAS scores will be recorded at rest and during movement at 2, 4, 6, 8, 12, 24, and 48 h postoperatively. The time of the first pressing of the analgesic pump will be documented. The rescue analgesia rate and analgesic satisfaction scores will be assessed at 24 and 48 h postoperatively. Postoperative satisfaction will be evaluated (0 = dissatisfied, 1 = average, 2 = satisfied, and 3 = very satisfied). Postoperative awakening and extubation time will be recorded. The extubation criteria are that the patient resumes spontaneous breathing with a tidal volume ≥7 mL/kg, demonstrates swallowing reflex, and can follow instructions, maintaining SpO_2_ above 90%. The duration of the postoperative PACU stay and discharge time will be recorded. The occurrence rates of opioid-related side effects will be recorded, including dizziness and urinary retention. The serum NET levels will be measured. In this study, we will assess NETs levels indirectly by quantifying cell-free double-stranded DNA (cf-dsDNA) in serum using the PicoGreen dsDNA quantitation method. Serum samples (5 mL) are collected, centrifuged, and stored at −20 °C. The working dye solution is prepared by diluting PicoGreen reagent 1:200 in TE buffer. A standard curve is constructed using serial dilutions of calf thymus DNA. Equal volumes of diluted sample and dye are mixed and incubated in the dark at room temperature for 5 min. Fluorescence intensity is measured using a fluorescence microplate reader, and the cf-dsDNA concentration (ng/mL) is calculated using the standard curve. This measurement serves as an indirect indicator of NET formation in the postoperative period.

Throughout the study, data will be gathered and managed securely and privately. Data will be collected automatically via the anesthetic information system and vital sign monitoring system. A nursing anesthetist will also collect some data manually. Participants will be identified throughout the study using code numbers rather than names unless otherwise specified. All relevant records and files will be archived for 5 years. During the study, the data will be accessible only to researchers who have signed confidential disclosure agreements and institutional or government auditors. Data without patient identification will be accessible to the general public after the study concludes.

### 2.8 Outcomes measures

#### 2.8.1 Primary outcome measure

The primary outcomes include nausea and vomiting, antiemetic requirements in the PACU, and the first and second 24-h periods postoperatively, and include the neutrophil count, lymphocyte count, and NLR on the first postoperative day. Patients with a nausea and vomiting score ≥1 (0 = no nausea, 1 = nausea, 2 = retching, 3 = vomiting) will be treated with ondansetron as an antiemetic. Please refer to the attached nausea and vomiting scoring sheets.

#### 2.8.2 Secondary outcome measure

The secondary outcomes include the following variables: (1) VAS scores during rest and movement for patients at 2, 6, 12, 24, and 48 h postoperatively; (2) consumption of propofol, remifentanil, and other drugs during surgery, as well as the total consumption of analgesics in the first and second 24-h periods postoperatively; (3) the time of the first press on the patient-controlled analgesia pump, pain satisfaction scores at 24 and 48 h, postoperative recovery time, extubation time, PACU stay time, and postoperative discharge time; (4) the occurrence of opioid-related side effects such as dizziness and urinary retention; (5) the concentration of NETs in serum on the first postoperative day.

### 2.9 Patient and public involvement

There will be no patient or public involvement in the conduct, reporting, or dissemination of this research.

### 2.10 Participant timeline

The post-enrollment timeline starts when the patient enters the operating room and ends when the patient leaves the PACU. All relevant variables will be recorded by individuals who are unaware of the grouping. The follow-up staff will evaluate the patient’s postoperative nausea and vomiting 48 h after the surgery to assess the relevant situation (Table 1).

**Table 1:** Study flow chart.

**Timepoint**	**-D1**	**-D1**	**D01**	**D02**	**PACU**	**2 h**	**6 h**	**12 h**	**24 h**	**48 h**	**D03**
Enrollment											
Patient screening	√										
Informed consent	√										
Allocation		√									
Random assignment		√									
Intervention:											
[EA group]			√								
[CA group]			√								
[EB group]			√								
[CB group]			√								
Assessments:											
Eligibility criteria		√									
Baseline data		√									
[Vital signs]		√									
[ESPB and assessment]			√								
[NLR]		√							√		
[PONV score]					√	√	√	√	√	√	
[Antiemetic requirements]					√	√	√		√	√	
[VAS score]					√	√	√	√	√	√	
[Consumption of propofol or remifentanil or analgesics]				√	√	√	√	√	√	√	
[The first pressanalgesia pump]					√	√	√	√	√	√	
[Pain satisfaction scores]					√	√	√		√	√	
[Extubation time]					√						
[PACU stay time]					√						
[NETs]									√		
[Postoperative discharge time]											√
Occurrence of adverse reactions:						√	√	√	√	√	
[Dizziness]					√	√	√	√	√	√	
[Urinary retention]					√	√	√	√	√	√	
[Other adverse reactions]					√	√	√	√	√	√	

-D1, The day before surgery; D01, Day of surgery, before the start of the operation; D02, During surgery; D03, Postoperative discharge time; ESPB, erector spinae plane block; NLR, neutrophil to lymphocyte ratio; PONV, postoperative nausea and vomiting; NETs, neutrophils form neutrophil extracellular traps.

### 2.11 Sample size calculation

To calculate the sample size for the primary research objective, use the “Tests for Two Ordered Categorical Variables” module in PASS software. Set the test power to 1 − β = 0.8 and significance level *α* = 0.05; use a 1:1 ratio between the NLR < 2 and NLR ≥ 2 groups. For the NLR < 2 group, the composition ratios of the PONV states considered as no, nausea, retching, and vomiting are 0.575, 0.050, 0.001, and 0.375, respectively. For the NLR ≥ 2 group, the composition ratios for PONV no, nausea, retching, and vomiting are 0.250, 0.100, 0.050, and 0.600, respectively. The calculated sample size is 94 cases, considering a 10% dropout rate. The final sample size is 106, with 53 patients in each group.

Use the “Tests for Two Proportions” module in PASS software to calculate the sample size for one of the secondary objectives: determining whether preoperative NLR can serve as a biomarker for PONV. Set the test power to 1 − β = 0.8 and significance level to *α* = 0.05; use a 1:1 ratio between the control and thoracic paravertebral nerve block groups. Nausea and vomiting rates are 8.3 and 33.3%, respectively. The calculated sample size is 82, considering a 10% dropout rate. The final sample size is 92, with 46 patients in each group.

The baseline NLR levels are first divided into two strata, and randomization is performed within each stratum. The sample size of each group in this study is 55.

### 2.12 Randomization and blinding

Patients are grouped using block randomization; drug blinding is conducted by specialized personnel who are not directly involved in the clinical trial. Block randomization will be performed using random number tables generated by the SAS statistical software.

This trial is designed to be double-blinded; both the participants and follow-up personnel will be unaware of the group assignments. The overall randomization table is maintained by the personnel responsible for the statistics. The researchers prepare the drugs using an overall randomization table. Postoperative follow-up is conducted by anesthesia nurses who have received specialized training. Unblinding is conducted according to the normal procedures if no emergencies occur during the study. After the trial concludes, the Clinical Report Form (CRF) will be checked. Following signatures, unblinding will be performed to determine the group assignments of the study participants for statistical analysis and evaluation of efficacy differences between the experimental and control groups. Emergency unblinding is allowed only when the study drug affects the treatment or safety of the study participants. The principal investigator will be notified, and emergency unblinding will be performed based on the drug information provided in the emergency letter. This information will be noted in the CRF.

### 2.13 Statistical analysis

All data will be statistically analyzed using SPSS software (version 25.0). Continuous variables such as VAS scores will be represented by the mean ± standard deviation if they follow a normal distribution. An independent sample t-test will be employed to compare intergroup differences. If the data do not conform to a normal distribution, the Wilcoxon rank-sum test will be used to compare groups.

For categorical variables, such as sex, the chi-squared or Fisher’s exact test will be used to assess intergroup differences. For ordinal data, such as the degree of vomiting, the Wilcoxon rank-sum test will be used for between-group comparisons.

## References

[1] Apfel CC Heidrich FM Jukar-Rao S Jalota L Hornuss C Whelan RP . Evidence-based analysis of risk factors for postoperative nausea and vomiting. Br J Anaesth. (2012) 109:742–53. doi: 10.1093/bja/aes276, PMID: 23035051

[2] Gan TJ Diemunsch P Habib AS Kovac A Kranke P Meyer TA . Consensus guidelines for the Management of Postoperative Nausea and Vomiting. Anesth Analg. (2014) 118:85–113. doi: 10.1213/ANE.0000000000000002, PMID: 24356162

[3] Moreno C Veiga D Pereira H Martinho C Abelha F. Postoperative nausea and vomiting: incidence, characteristics and risk factors--a prospective cohort study. Rev Esp Anestesiol Reanim. (2013) 60:249–56. doi: 10.1016/j.redar.2013.02.005, PMID: 23582584

[4] Williams KS. Postoperative nausea and vomiting. Surg Clin N Am. (2005) 85:1229–41. doi: 10.1016/j.suc.2005.09.005, PMID: 16326204

[5] Phillips C Brookes CD Rich J Arbon J Turvey TA. Postoperative nausea and vomiting following orthognathic surgery. Int J Oral Maxillofac Surg. (2015) 44:745–51. doi: 10.1016/j.ijom.2015.01.006, PMID: 25655765 PMC4430405

[6] Arpaci AH Isik B Ilhan E Erdem E. Association of Postoperative Nausea and Vomiting Incidence with neutrophil-lymphocyte ratio in ambulatory maxillofacial surgery. J Oral Maxillofac Surg. (2017) 75:1367–71. doi: 10.1016/j.joms.2016.12.036, PMID: 28137634

[7] Azab B Daoud J Naeem FB Nasr R Ross J Ghimire P . Neutrophil-to-lymphocyte ratio as a predictor of worsening renal function in diabetic patients (3-year follow-up study). Ren Fail. (2012) 34:571–6. doi: 10.3109/0886022X.2012.668741, PMID: 22452450

[8] Wilson JM Lohser J Klaibert B. Erector spinae plane block for postoperative rescue analgesia in Thoracoscopic surgery. J Cardiothorac Vasc Anesth. (2018) 32:e5–7. doi: 10.1053/j.jvca.2018.06.026, PMID: 30093190

[9] Chin KJ Adhikary S Sarwani N Forero M. The analgesic efficacy of pre-operative bilateral erector spinae plane (Esp) blocks in patients having ventral hernia repair. Anaesthesia. (2017) 72:452–60. doi: 10.1111/anae.13814, PMID: 28188621

[10] Forero M Adhikary SD Lopez H Tsui C Chin KJ. The erector spinae plane block: a novel analgesic technique in thoracic neuropathic pain. Reg Anesth Pain Med. (2016) 41:621–7. doi: 10.1097/AAP.0000000000000451, PMID: 27501016

[11] Forero M Rajarathinam M Adhikary SD Chin KJ. Erector spinae plane block for the Management of Chronic Shoulder Pain: a case report. Can J Anaesth. (2018) 65:288–93. doi: 10.1007/s12630-017-1010-1, PMID: 29134518

[12] Forero M Rajarathinam M Adhikary S Chin KJ. Erector spinae plane (Esp) block in the Management of Post Thoracotomy Pain Syndrome: a case series. Scand J Pain. (2017) 17:325–9. doi: 10.1016/j.sjpain.2017.08.013, PMID: 28919152

[13] Melvin JP Schrot RJ Chu GM Chin KJ. Low thoracic erector spinae plane block for perioperative analgesia in lumbosacral spine surgery: a case series. Can J Anaesth. (2018) 65:1057–65. Epub 2018/04/29. doi: 10.1007/s12630-018-1145-8, PMID: 29704223

[14] Chan AW Tetzlaff JM Gotzsche PC Altman DG Mann H Berlin JA . Spirit 2013 explanation and elaboration: guidance for protocols of clinical trials. BMJ. (2013) 346:e7586. doi: 10.1136/bmj.e7586, PMID: 23303884 PMC3541470

[15] Watcha MF White PF. Economics of Antiemetics in anesthesia. Curr Opin Anaesthesiol. (2001) 14:563–7. doi: 10.1097/00001503-200110000-00018, PMID: 17019148

[16] Wittermans E van de Garde EM Voorn GP Aldenkamp AF Janssen R Grutters JC . Neutrophil count, lymphocyte count and neutrophil-to-lymphocyte ratio in relation to response to adjunctive dexamethasone treatment in community-acquired pneumonia. Eur J Intern Med. (2022) 96:102–8. doi: 10.1016/j.ejim.2021.10.030, PMID: 34782191

[17] Apfel CC Kranke P Katz MH Goepfert C Papenfuss T Rauch S . Volatile anaesthetics may be the main cause of early but not delayed postoperative vomiting: a randomized controlled trial of factorial design. Br J Anaesth. (2002) 88:659–68. doi: 10.1093/bja/88.5.659, PMID: 12067003

[18] Apfel CC Greim CA Haubitz I Grundt D Goepfert C Sefrin P . The discriminating power of a risk score for postoperative vomiting in adults undergoing various types of surgery. Acta Anaesthesiol Scand. (1998) 42:502–9. Epub 1998/05/30. doi: 10.1111/j.1399-6576.1998.tb05158.x, PMID: 9605364

[19] Ali-Melkkila T Kanto J Katevuo R. Tropisetron and metoclopramide in the prevention of postoperative nausea and vomiting. A comparative, placebo controlled study in patients undergoing ophthalmic surgery. Anaesthesia. (1996) 51:232–5. doi: 10.1111/j.1365-2044.1996.tb13639.x, PMID: 8712322

[20] Chan MT Chui PT Ho WS King WW. Single-dose Tropisetron for preventing postoperative nausea and vomiting after breast surgery. Anesth Analg. (1998) 87:931–5. doi: 10.1097/00000539-199810000-00035, PMID: 9768797

[21] Tayfur C Burcu DC Gulten O Betul D Tugberk G Onur O . Association between platelet to lymphocyte ratio, Plateletcrit and the presence and severity of hyperemesis Gravidarum. J Obstet Gynaecol Res. (2017) 43:498–504. doi: 10.1111/jog.13228, PMID: 28160526

[22] Bolignano D Lacquaniti A Coppolino G Donato V Campo S Fazio MR . Neutrophil gelatinase-associated Lipocalin (Ngal) and progression of chronic kidney disease. Clin J Am Soc Nephrol. (2009) 4:337–44. doi: 10.2215/CJN.03530708, PMID: 19176795 PMC2637601

[23] Hong J Mao Y Chen X Zhu L He J Chen W . Elevated preoperative neutrophil-to-lymphocyte ratio predicts poor disease-free survival in Chinese women with breast Cancer. Tumour Biol. (2016) 37:4135–42. doi: 10.1007/s13277-015-4233-1, PMID: 26490984

[24] Ji F Liang Y Fu SJ Guo ZY Shu M Shen SL . A novel and accurate predictor of survival for patients with hepatocellular carcinoma after surgical resection: the neutrophil to lymphocyte ratio (Nlr) combined with the aspartate aminotransferase/platelet count ratio index (Apri). BMC Cancer. (2016) 16:137. doi: 10.1186/s12885-016-2189-1, PMID: 26907597 PMC4763424

[25] Jung MR Park YK Jeong O Seon JW Ryu SY Kim DY . Elevated preoperative neutrophil to lymphocyte ratio predicts poor survival following resection in late stage gastric Cancer. J Surg Oncol. (2011) 104:504–10. doi: 10.1002/jso.21986, PMID: 21618251

[26] Kucuk A Erol MF Senel S Eroler E Yumun HA Uslu AU . The role of neutrophil lymphocyte ratio to leverage the differential diagnosis of familial Mediterranean fever attack and acute appendicitis. Korean J Intern Med. (2016) 31:386–91. doi: 10.3904/kjim.2015.039, PMID: 26864298 PMC4773722

[27] Lou M Luo P Tang R Peng Y Yu S Huang W . Relationship between neutrophil-lymphocyte ratio and insulin resistance in newly diagnosed type 2 diabetes mellitus patients. BMC Endocr Disord. (2015) 15:9. doi: 10.1186/s12902-015-0002-9, PMID: 25887236 PMC4357061

[28] Qin B Ma N Tang Q Wei T Yang M Fu H . Neutrophil to lymphocyte ratio (Nlr) and platelet to lymphocyte ratio (Plr) were useful markers in assessment of inflammatory response and disease activity in Sle patients. Mod Rheumatol. (2016) 26:372–6. doi: 10.3109/14397595.2015.1091136, PMID: 26403379

[29] Uslu AU Kucuk A Sahin A Ugan Y Yilmaz R Gungor T . Two new inflammatory markers associated with disease activity Score-28 in patients with rheumatoid arthritis: neutrophil-lymphocyte ratio and platelet-lymphocyte ratio. Int J Rheum Dis. (2015) 18:731–5. doi: 10.1111/1756-185X.12582, PMID: 25900081

